# Cerebro-Spinal Meningitis

**Published:** 1875-05

**Authors:** William Reed

**Affiliations:** Boston, Mass.


### CEREBRO-SPINAL MENINGITIS.

BY WM. READ, M. D., BOSTON, MASS.

September 2d, 1874. Lind, æt. twenty-nine. Patient is by occupation a teacher of gymnastics, and is usually in the enjoyment of robust health. Was perfectly well on Sunday last (three days ago), but during the night began to be sick. Had a chill. Next day (Monday) headache came on, and with it a tendency to opisthotonos. Has been growing worse all the time. Now, 10 A. M., in bed; excited; mind wandering and full of hallucinations. Pulse 72, irregular, but full. Complains of a sharp pain through the head at the temples, in front of the ears, and at the very top. Skin moist; feet and legs cold; face slightly flushed; answers questions correctly, but immediately wanders and talks at random. Spine extremely tender from the nuchæ to the middle of the dorsal region. At a point opposite spine of scapula has frequent spasms of opisthotonic distortion, during which the head is thrown very far back, and her limbs are convulsed. Is with difficulty kept on the bed, requiring two or three to hold her. Had several of these paroxysms during my visit; complains of a feeling of great prostration; bowels well; breath rather offensive.

To have a full dose of castor oil mixed with an equal amount of glycerine, at once, and then—℞. Ergotine, gr. x; ext. belladonnæ, gr. j.; M. Ft. pil. x. Sig. Take one every three hours.

Croton oil to be applied to the nuchæ till a copious eruption is produced.

September 3d. 10 A. M. Has slept most of the time since last record. Complains all the time of feeling "so tired." Has had no opisthotonos since yesterday morning, but starts frequently in her sleep. Rational; skin normal; action of iris undiminished; tongue well; relishes her nourishment, a gill of milk porridge and of beef tea alternately every three hours. Position in bed natural. Pulse 52. Not irregular at all, but of marked variation in strength and fullness. Extremities warm; less headache, and the pain in her spine is less severe than it was. Is for the most part drowsy, but conscious of the slightest sudden noise. Continued treatment.

September 4th, A. M. Has been very comfortable with no new symptoms. No true sleep until 4 A. M. to-day. Since then dozing quietly. Has had no spasms, and complains less of headache. Extremities warm; urine very scanty and high colored; tongue slightly coated; breath quite offensive; still feels "so tired;" takes her nourishment well; occasionally twitches a little when asleep; has an uncomfortable feeling in the spine, not exactly a pain, but a "creeping feeling." Has had this feeling all along, and thinks it is growing less. Hearing somewhat obscured; eyesight well; is naturally very near-sighted. Continued treatment, and have spts. ether nit. ʒj. in water, p. r. n.

September 5th. Had a very comfortable day, till the evening, when some friends called, and fatigued her by talking. During the first part of the night she got some sleep, and was quiet. Towards morning patient began to have attacks of momentary spasm, or starting, whether she was asleep or awake, but has at no time lost her consciousness. About the same time with the commencement of these attacks a chill came on. Now, 10 A. M., pulse 58, of fair volume and regular; tongue quite clean; pupils perfectly mobile and unaffected by the bellad; hearing about the same as at last record; no petechiæ; her whole body is in a state of hyperæsthesia.

The slightest touch of the hand causes pain, which goes away on deeper pressure, except along the spine and just above the crest of the ilium, on either side. The spine is very tender to the touch, the whole length, from the nuchæ to the os cocygis. Says her head is better; she has less pain and uneasiness about it than two days ago, while there is more of the "creeping feeling." Continued treatment, and paint the spine with a liquid blistering preparation (acetic cantharidal vesicant).

September 6th, A. M. Slept well and quietly all night; blister did not draw fully; head much more easy and free from pain; hearing quite good; eyesight well, in the right; the left eye was somewhat inflamed, but it has disappeared. Tongue well; the starting in her sleep has nearly ceased; hyperæsthesia almost gone; has had no movement of the bowels for two days; spine feels easier; urine is still scanty in quantity, but of lighter color. Pulse 58. No petechiæ to be seen. Have a cathardic dose of ol. ric. and glycerine.

September 7th. Had four dejections from the cathartic ordered yesterday; slept well; has none of the "creeping feeling" in her spine, and can lie on her back without suffering. Head quite free from ache; hearing improves daily; no petechiæ; there is no hyperæsthesia remaining, but on the inside of the legs and arms, along the course of the great veins, there is decided tenderness. Pulse 60, of good healthy fullness. Tongue slightly coated; eyes act freely with the light.

September 8th. Has been quite comfortable. Some headache in the P. M., but not enough to cause much discomfort. When lying still has no uncomfortable feeling at all in her spine, and only a slight uneasiness when she turns over. Sleeps a good deal; does not feel hungry, although she takes her nourishment well. Pulse 60, of normal volume. The tenderness along the inside of the thighs, from the groin as far down as the knee joint, remains the same. It has disappeared from the arms; hyperæsthesia entirely gone. Continued treatment.

September 9th. Reports herself entirely free from pain everywhere. There is still some pain along the inside of both thighs, but it is diminished. Tongue more coated than at any previous day; appetite is coming back. As patient lies in bed, says she feels as well as ever she did. Diminish the ergot and belladonna to two doses daily.

September 10th. Patient continues to improve. When perfectly quiet, feels well. The spine is a little tender over one of the dorsal vertebræ, a small space in the lumbar region and at the coccyx. In the inside of the left thigh there still remains some tenderness, but along the right thig hit has almost entirely disappeared. Tongue clean; appetite good. Continued treatment as last ordered.

September 11th. Much better. Is partially dressed and lying on the lounge. Continued treatment.

September 12th. No tenderness along the spine. No uncomfortable feeling about the head. There still remains a slight tenderness about six inches above the knee, on the inside of the left thigh, but on the right it is entirely gone. Omit medicine.

September 14th. Walked into the next room this A. M., but on returning felt quite faint, and had to lie down. Says her head ached yesterday after talking a good deal. The only remaining symptoms are two spots of tenderness on the inside of the left thigh. One mentioned in yesterday's report, and the other about three inches higher up.

September 17th. Since last record has been steadily gaining.—Tenderness entirely gone from the left thigh; pulse normal in frequency and character; appetite good. The only disability she suffers from is the weakness resulting from the attack. To have tr. gent. co. ℨij, twice a day as a tonic. Ceased attendance.—*Med. & Surg. Reporter.*

